# Independence

**Published:** 1874-10

**Authors:** 


### INDEPENDENCE.

It was an agreeable surprise to all observant persons going to the Vienna Exhibition to be able to find in the hotels of Switzerland and Germany so many young men who could speak English fluently, and who, by their tasteful dress and easy manners, and intelligent conversation, showed that they were far above the menial class. The question was, how did they acquire such a facility in speaking English. The general answer was, that during the winter they went to England and acted as waiters in the hotels and restaurants, where their services were very valuable as speaking German and French; thus in the course of a very few years they speak several foreign languages with fluency, and thus become quite an acquisition to mercantile and banking establishments, to be themselves merchants and bankers. At the Twin Mountain House, near the foot of Mount Washington, there was a young lady waiter whose dress and manner and conversation was very considerably in advance of the same class in New York. In conversation with her it was elicited that at home she had her own piano, was an accomplished performer, and had educated herself up to a position most creditable to her energy, self-reliance and independence, by getting good wages at hotels during the summer months.

It is creditable to the young gentlemen who attend some of our New England colleges that they have followed the example of the young ladies, and have scattered themselves among the mountains of New Hampshire and other summer resorts to act as waiters, coachmen and clerks, and boys of all work, willing to groom a horse or black a boot, or brush a coat whenever it comes in the way. This is true independence. Such young men will make their way in the world, and are destined to occupy positions of high responsibility in the future. Their very self-assertion insures their success. Suppose our "beneficiary" theological students take a hint in this direction, and instead of idling away their summer vacations in visiting, and lounging about home as gentlemen loafers, they would not only be able to earn forty or fifty, or a hundred dollars during the season, but would learn to make themselves "handy," useful in the household when they become married men, useful to their wives and families in a hundred little ways, in cases of "company," of sickness, and the sudden leaving of the "help," and thus relieve the often overburdened wife of much of her added labor.

